# Investigating the discernible and distinct effects of platinum-based chemotherapy regimens for metastatic triple-negative breast cancer on time to progression

**DOI:** 10.3892/ol.2014.1782

**Published:** 2014-01-07

**Authors:** DANIEL KHALAF, JOHN F. HILTON, MARK CLEMONS, LAURENT AZOULAY, HUI YIN, LISA VANDERMEER, SUSAN DENT, SEAN HOPKINS, NATHANIEL BOUGANIM

**Affiliations:** 1 Division of Hematology-Oncology, University of Montreal - Notre-Dame Hospital, Montreal, QC H2L 4M1, Canada; 2 Division of Medical Oncology, The Ottawa Hospital Cancer Centre, Ottawa, ON K1H 8L6, Canada; 3 Department of Oncology, Royal Victoria Hospital, McGill University, Montreal, QC H3A 1A1, Canada; 4 Centre for Clinical Epidemiology, Lady Davis Institute, Jewish General Hospital, Montreal, QC H3T 1E2, Canada; 5 Department of Pharmacy, The Ottawa Hospital Cancer Centre, Ottawa, ON K1H 8L6, Canada

**Keywords:** triple-negative breast cancer, time to progression, metastatic breast cancer, platinum

## Abstract

Platinum-based chemotherapy regimens are frequently used in patients with triple-negative breast cancer (TNBC). The aim of the current study was to assess whether or not platinum-based chemotherapy is associated with an increased time to progression when compared with non-platinum-based regimens in TNBC and non-TNBC. A retrospective analysis was conducted within a cohort of patients with metastatic breast cancer who received platinum-based chemotherapy at a single institution. Data were collected for up to three lines of treatment for metastatic disease. Time to progression was determined for platinum-based chemotherapy and non-platinum-based regimens for each line of treatment. Adjusted hazard ratios (HRs), together with 95% confidence intervals (CIs) were estimated comparing the time to progression associated with the use of platinum-based chemotherapy versus non-platinum-based regimens. A total of 159 patients were included in the analysis, with 58 diagnosed with TNBC. Among the patients with TNBC, compared with non-platinum-based chemotherapy, no correlation was identified between platinum-based chemotherapy and an improved time to progression [first line: HR, 0.97 (95% CI, 0.40–2.35); second line: HR, 0.91 (95% CI, 0.42–2.01); and third line: HR, 2.83 (95% CI, 0.73–11.03)]. By contrast, patients with non-TNBC appeared to improve with non-platinum-based chemotherapy [first line: HR, 2.57 (95% CI, 1.11–5.99); second line: HR, 1.91 (95% CI, 1.00–3.63); and third line: HR, 1.08 (95% CI, 0.53–2.18)]. Although the present study was limited by the sample size and its observational nature, the results indicated that platinum-based chemotherapy does not offer a discernible or distinct advantage compared with standard regimens in patients with TNBC, and is perhaps less efficacious in patients with non-TNBC.

## Introduction

Previously, platinum-based chemotherapy regimens were not commonly prescribed for patients with metastatic breast cancer, as other regimens were considered to exhibit improved efficacy and toxicity profiles (1). Interest in platinum-based therapies was renewed with the observation that BRCA1 deficient cell lines have a higher sensitivity to DNA crosslinking agents, such as cisplatin, compared with other breast cancer cell lines (2). There also appears to be a significant overlap in terms of the histological and molecular features between BRCA1-deficient breast tumors and types of triple-negative breast cancer (TNBC) (3). Due to these similarities, it has been hypothesized that the DNA repair defects that sensitize BRCA1-deficient breast cancer tumors to platinum may also be present in TNBC, indicating that platinum-based chemotherapies may be an effective treatment option for this subset of breast cancer (4).

While the exact role of platinum-based chemotherapies in TNBC is being explored (Triple-Negative Trial, NCT00532727; http://www.clinicaltrials.gov), there appears to be conflicting data from retrospective studies with regard to efficacy in the neoadjuvant (5) and metastatic settings (6). Previously, in two prospective neoadjuvant studies, the use of single agent cisplatin in patients with TNBC resulted in pathological complete response rates of 22 (7) and 10% (8), respectively. In the metastatic setting, a multicenter phase II trial tested cisplatin or carboplatin first-line chemotherapy for patients with metastatic TNBC. Although there was a response rate of 30%, the median progression-free survival time was only 3 months (9). These results are not superior to historical data that shows that chemotherapy for triple-negative disease has a median response duration of 12, 9 and 4 weeks in first-, second- and third-line settings, respectively (10). Given this, it does not appear that platinum has any definite additional activity compared with that of the more commonly used regimens.

As the role of platinum-based chemotherapy in the treatment of TNBC is controversial, the present study reviewed platinum-based chemotherapy in metastatic breast cancer at the Ottawa Hospital Cancer Center (Ottawa, Canada). The main objective was to assess whether platinum-based chemotherapy is more effective than non-platinum-based chemotherapy in patients with metastatic TNBC in terms of time to progression. Similarly, it was assessed whether platinum-based chemotherapy is more effective than non-platinum-based chemotherapy in metastatic non-TNBC.

## Patients and methods

### Patient characteristics

All patients with histologically confirmed metastatic or locally recurrent breast cancer who received platinum-based chemotherapy at the Ottawa Hospital Cancer Centre (Ottawa, Canada) between January 2000 and September 2010 were identified. Patient data were collected through a review of electronic health records.

Patients were separated into two cohorts, TNBC and non-TNBC. Patients were classified as TNBC based on their surgical or biopsy results, which were defined as follows: i) Estrogen and progesterone receptor levels <1% by immunohistochemistry (IHC); and ii) human epidermal growth factor receptor 2 scored as 0, 1 or 2 on IHC and/or negative fluorescence *in situ* hybridization testing (11). Eligible patients were required to have received any single or combination drug platinum-based chemotherapy regimen for incurable disease. Patients with an incomplete receptor status were excluded, as well as those who had not received platinum specifically for advanced disease or those who had received chemotherapy for separate types of concomitant cancer. The present study received Research Ethics Board approval from the Ottowa Hospital Cancer Center.

The reason for discontinuation of every line of chemotherapy (e.g. toxicity and disease progression) and the date of disease progression were determined from the clinical notes. The primary outcome was time to progression, defined from the start date of one line of chemotherapy to the date of the last cycle administered prior to documented disease progression, clinical deterioration with no further chemotherapy or documented mortality. Lines of chemotherapy with an unclear outcome due to loss to follow-up were not considered in the analysis.

### Statistical analysis

Descriptive statistics were used to summarize the characteristics of the patients with TNBC and non-TNBC. For each study cohort (TNBC and non-TNBC), Kaplan-Meier curves were constructed comparing the cumulative incidence of disease progression for patients exposed to platinum-based chemotherapy versus non-platinum-based chemotherapy. Differences between curves were assessed by calculating log-rank test P-values. P<0.05 was considered to indicate a statistically significant difference. In addition, crude incidence rates of disease progression were calculated for platinum-based chemotherapy and non-platinum-based chemotherapy, together with 95% confidence intervals (CIs) based on the Poisson distribution. Cox proportional hazards models were used to estimate crude and adjusted hazard ratios (HRs) and 95% CIs of disease progression associated with the use of platinum-based chemotherapy versus non-platinum-based chemotherapy for each line of chemotherapy. Under this scheme, various models were constructed for each line of chemotherapy and thus, it was possible for patients to contribute data to more than one line of chemotherapy. The models were adjusted for age, prior adjuvant and neoadjuvant chemotherapy, previous use of platinum (in models of second- and third-line chemotherapy), tumor grade, initial stage, site and extent of first distant relapse and presence of brain metastasis. Results were analyzed using SAS version 9.2 (SAS Institute Inc., Cary, NC, USA).

## Results

### Patient population

A total of 173 patients with metastatic or locally recurrent breast cancer received platinum-based chemotherapy. In total, 14 patients were excluded due to incomplete receptor status results, leaving 58 patients in the TNBC cohort and 101 patients in the non-TNBC cohort. Of these, 50 patients in each cohort received platinum-based chemotherapy in the first, second or third line. Due to the anticipated shorter survival of the TNBC cohort, comparisons for this study were restricted to the first three lines of therapy.

Baseline patient characteristics for each group are shown in Table I. Prior adjuvant chemotherapy was received by 55 and 66% of TNBC and non-TNBC patients, respectively, and the rates of prior neoadjuvant chemotherapy were 36 and 31%, respectively. At the onset of metastatic disease, 59% of the patients with TNBC exhibited visceral metastasis, including 14% with initial brain metastasis, compared with 53% of non-TNBC patients who exhibited visceral metastasis, including 5% with initial brain metastasis. By the end of study period, 87% of the entire cohort had succumbed to their diseases or were receiving no further anticancer treatment. In addition, 5% of patients were lost to follow-up.

Table II shows the types of platinum-based regimens used. The combinations of vinorelbine or gemcitabine with cisplatin or carboplatin were the most frequent platinum-based regimens used (>70%). On average, TNBC patients received 2.8 lines of chemotherapy in the metastatic setting (range, 1–5) versus 3.9 lines for non-TNBC patients (range, 1–6). TNBC patients received platinum in the first, second or third lines in 27, 36.5 and 17.6% of cases, respectively, compared with 12.4, 21.2 and 20.9% of cases, respectively, for the non-TNBC cohort.

### Disease progression

**Figure 1:**
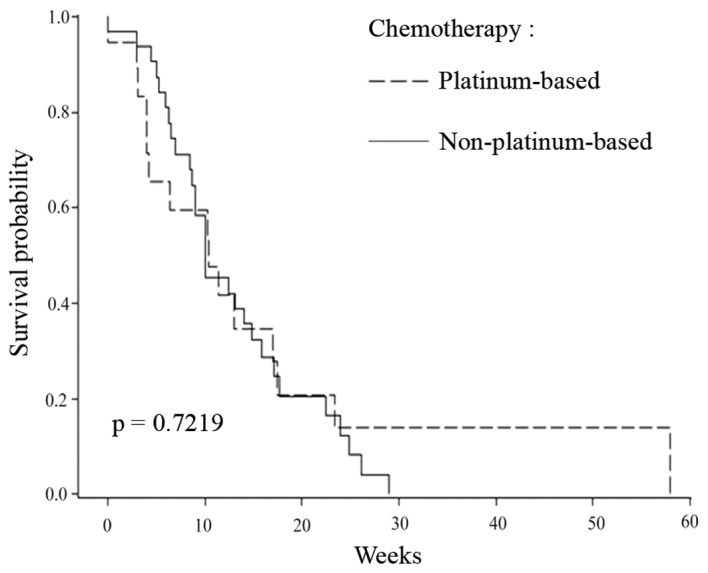
Kaplan-Meier estimate of disease progression for first-line chemotherapy of the TNBC cohort. TNBC, triple-negative breast cancer.

**Figure 2:**
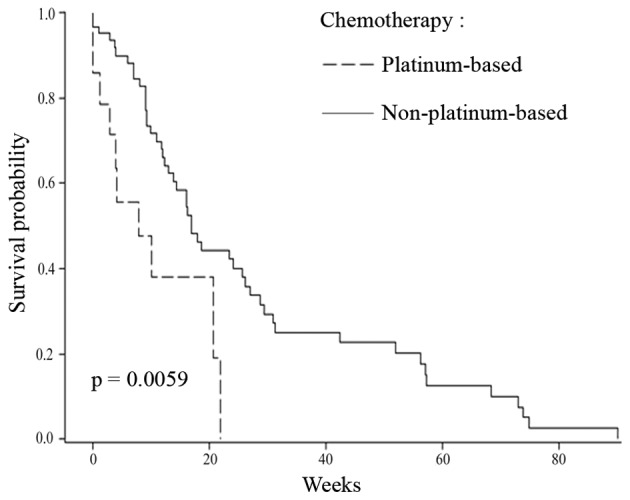
Kaplan-Meier estimate of disease progression for first-line chemotherapy of the non-TNBC cohort. TNBC, triple-negative breast cancer.

With respect to the cumulative incidence of disease progression, no statistically significant differences were observed between the use of platinum-based chemotherapy and non-platinum-based regimens as first-line chemotherapy in patients with TNBC (Fig. 1). By contrast, in the non-TNBC cohort, patients who received platinum exhibited a poorer time to progression when administered in the first-line setting (Fig. 2).

In the two cohorts, the main reason for the discontinuation of chemotherapy was disease progression and a higher proportion of patients discontinued chemotherapy due to toxicity in the TNBC cohort compared with the non-TNBC cohort (17.1, vs. 8.9%). The median overall survival time from the time of the diagnosis of metastatic disease was 60 weeks for the TNBC cohort and 144 weeks for the non-TNBC cohort.

Table III presents the results comparing platinum-based chemotherapy with non-platinum-based regimens in the first-, second- and third-line settings in patients with TNBC. Overall, the use of platinum-based chemotherapy was not found to correlate with an improved time to progression, with the adjusted HRs close to unity in the first- and second-line settings. The adjusted HR for the third-line setting was numerically elevated and did not reach statistical significance, although the point estimate was likely unstable due to the few patients in this group.

For patients with non-TNBC, the use of platinum-based chemotherapy regimens in the first-line setting was associated with a >2-fold increased risk in disease progression (HR, 2.57; 95% CI, 1.11–5.99). In the second- and third-line settings, the use of platinum-based chemotherapy regimens was not found to correlate with disease progression (HR, 1.08; 95% CI, 0.53–2.18 and HR, 1.91; 95% CI, 1.00–3.63, respectively).

## Discussion

Despite the aggressive phenotype, there remains no standard chemotherapy regimen (12) for females with metastatic TNBC. This is important from two standpoints; firstly, the ‘best’ chemotherapy must be provided upfront for all patients regardless of their specific phenotype. An ongoing UK randomized, phase III Triple-Negative Breast Cancer Trial (TNT; NCT00532727) comparing single agent carboplatin with docetaxel for metastatic TNBC is likely to provide further information concerning the optimal treatment options for these patients. Secondly, in the absence of a ‘standard chemotherapy backbone’ it is difficult to know which chemotherapy regimens to add additional agents to in this patient population. For example, studies with poly (ADP-ribose) polymerase (13), epidermal growth factor receptor (14) and vascular endothelial growth factor (15,16) inhibitors have all used various chemotherapy backbones.

In the TNBC cohort of the present study, no observed benefit was identified in time to progression with the use of platinum-based chemotherapy compared with non-platinum-based regimens. Platinum-based chemotherapy was discontinued significantly more often due to toxicity and this must be factored in when considering their use. The current study was unable to determine whether platinum-based chemotherapy had an impact on survival in TNBC as every patient received a platinum-based chemotherapy at specific times. In addition, overall treatment for patients within the population was extremely heterogeneous, confounding any potential survival evaluation.

In the present study, patients with non-TNBC who received platinum-based regimens exhibited a shorter time to progression compared with those who received non-platinum-based regimens, particularly in the first-line setting. No previous randomized controlled trials have compared platinum-based chemotherapy with non-platinum-based chemotherapy in advanced breast cancer. Previously, in two small trials, single-agent cisplatin showed response rates of 47 and 54%, respectively, as a first-line treatment for advanced breast cancer (17,18). In phase II trials of combinations of carboplatin and taxanes as a first-line treatment for advanced breast cancer, response rates of 53–68% have been achieved (19). The results of the current study indicate that platinum-based chemotherapy may be inferior to non-platinum-based chemotherapy in non-TNBC.

The current study had limitations; firstly, as with any observational study, residual confounding may have been present. Considering that the population was a subset of patients observed at the cancer center who had received platinum-based therapy, there is the potential for a selection bias, which may affect the validity of the results. Secondly, the overall sample size was small and thus, the study was likely underpowered for the second- and third-line analyses, where the predicted benefits of chemotherapy greatly diminished. In addition, considering that chemotherapy is less effective in later lines of treatment compared with earlier lines, small differences between platinum- and non-platinum-based regimens may not have been detected in analysis. Finally, time to progression was not based on the Response Evaluation Criteria In Solid Tumors criteria and hence, a more pragmatic time to progression calculation was required.

Despite study limitations, the results of the current study indicate that platinum-based chemotherapy is not the ‘magic bullet’ for TNBC and that it appears to offer no significant advantage compared with more standard non-platinum-based regimens. In addition, platinum-based chemotherapy appears to correlate with a higher rate of discontinuation due to toxicity compared with other regimens. By contrast, platinum-based chemotherapy may be less effective compared with non-platinum-based regimens in patients with non-TNBC, although this may not be ascertained with any certainty from the current study. The prospective, randomized TNT is likely to aid the clarification of the role of platinum-based regimens in TNBC. The results of the current study are not consistent with the optimism for the widespread adoption of platinum-based chemotherapy in clinical practice.

## Figures and Tables

**Table I:** Baseline characteristics of TNBC and non-TNBC cohorts.

Characteristics	TNBC	Non-TNBC
Cohort size, n	58	101
Age at diagnosis, years (SD)	48.9 (11.2)	50.3 (9.8)
Time between diagnosis and 1st treatment, weeks (SD)	9.7 (21.4)	28.5 (59.4)
Extent of first distant relapse, n (%)^a^		
Single-site	39 (67.2)	61 (60.4)
Multi-site	19 (32.8)	40 (39.6)
Type of first distant relapse, n (%)^a^		
Non-visceral	24 (41.4)	47 (46.5)
Visceral	34 (58.6)	54 (53.5)
Adjuvant therapy, n (%)^a^		
No	18 (31.0)	26 (25.7)
Yes	32 (55.2)	67 (66.3)
Unknown	8 (13.8)	8 (7.9)
Neoadjuvant therapy, n (%)^a^		
No	36 (62.1)	68 (67.3)
Yes	21 (36.2)	32 (31.7)
Unknown	1 (1.7)	1 (1.0)
Disease metastatic to the brain, n (%)^a^		
At diagnosis	8 (13.8)	5 (4.9)
Following diagnosis	35 (60.3)	66 (65.4)
Unknown	15 (25.9)	30 (29.7)
Metastatic at diagnosis, n (%)^a^		
No	54 (93.1)	83 (82.2)
Yes	4 (6.9)	18 (17.8)
Median overall survival from diagnosis of metastatic disease, weeks	60	144
Patient status, n (%)		
Alive on treatment	4 (6.9)	6 (5.9)
Succumbed, unrelated to malignancy	1 (1.7)	1 (1.0)
Succumbed/palliative	49 (84.5)	90 (89.1)
Lost to follow-up	4 (6.9)	4 (4.0)

a Variables included in the adjusted model presented in Table III.

TNBC, triple-negative breast cancer.

**Table II:** Platinum-based regimens received.

Platinum regimens	Patients, n (%)
Cisplatin-vinorelbine	47 (26.4)
Carboplatin-vinorelbine	34 (19.1)
Cisplatin-gemcitabine	15 (8.4)
Carboplatin-gemcitabine	34 (19.1)
Cisplatin-etoposide	30 (16.9)
Other	18 (10.1)

**Table III:** Crude and adjusted HRs for time to progression associated with platinum-based regimens compared with standard chemotherapies for TNBC patients.

Treatment	Cases, n	Person-time (weeks)	Rate of progression (per 1,000/week)	Crude HR	Adjusted HR (95% CI)^a^
First line					
Standard chemotherapies	29	387	75.0	1.00	1.00 (reference)
Platinum-based chemotherapy	15	241	62.2	0.89	0.97 (0.40–2.35)
Second line					
Standard chemotherapies	18	416	43.3	1.00	1.00 (reference)
Platinum-based chemotherapy	22	599	36.7	0.81	0.91 (0.42–2.01)
Third line					
Standard chemotherapies	18	279	64.4	1.00	1.00 (reference)
Platinum-based chemotherapy	13	143	90.7	1.30	2.83 (0.73–11.03)

a Adjusted for the variables listed in Table I.

HRs, hazard ratios; CI, confidence interval; TNBC, triple-negative breast cancer.
